# Where you live matters: socioeconomic disparities in out-of-hospital cardiac arrest incidence and survival in Western Australia – A population-based cohort study

**DOI:** 10.1016/j.resplu.2026.101264

**Published:** 2026-02-18

**Authors:** Sarah Ann Harris, Stephen Ball, David Majewski, Jason Belcher, Judith Finn

**Affiliations:** a Prehospital, Resuscitation and Emergency Care Research Unit (PRECRU), Curtin School of Nursing, Curtin University, Bentley, Western Australia, Australia; b St John Western Australia, Western Australia, Australia; c Department of Epidemiology and Preventive Medicine, Monash University, Victoria, Australia; d Emergency Medicine, Medical School, The University of Western Australia, Perth, Australia

**Keywords:** Out-of-hospital cardiac arrest, Socioeconomic status, Epidemiology, Emergency medical service, Survival, Incidence

## Abstract

**Aims:**

Investigate socioeconomic differences in the incidence and odds of survival of adult out-of-hospital cardiac arrests [OHCA] in Western Australia [WA].

**Methods:**

OHCA data for patients aged 15 years and older were sourced for the period 2015–2024 for WA. Socioeconomic status was classified using the Australian Bureau of Statistics Index of Relative Socio-Economic Disadvantage. Socioeconomic status was attributed based on each patient’s residential address and classified in quintiles. Crude and age-standardised incidence rates per 100,000 adult population was calculated for all OHCA. Survival (return of spontaneous circulation at hospital arrival and survival to 30-days) was assessed for resuscitation-attempted OHCA with medical aetiology. Incidence by socioeconomic quintile was modelled using negative binomial linear regression, and odds of survival by socioeconomic quintile using logistic regression.

**Results:**

A total of 23,975 OHCAs were attended by emergency medical services in WA, with a crude incidence of 117.34 per 100,000 population per year. Outcomes varied across the socioeconomic gradient, with higher incidence and poorer survival in more disadvantaged populations. Incidence decreased from 193.81/100,000 in the most disadvantaged quintile to 73.18/100,000 in the least disadvantaged quintile (*p* < 0.001). Among resuscitation-attempted medical OHCA, patients in the least disadvantage areas had 67% greater odds of 30-day survival than those in the most disadvantaged areas (OR: 1.67; 95%CI: 1.28–2.18).

**Conclusion:**

Greater socioeconomic disadvantage is associated with a greater incidence of OHCA and poorer survival outcomes.

## Highlights

• OHCA incidence increased with increasing socioeconomic disadvantage.
• OHCA survival declined with increasing socioeconomic disadvantage.
• High SES patients had 67% greater odds of survival than low SES patients.
• SES was assigned using small-area (SA1) data, providing finer spatial resolution.

## Graphical abstract

**Figure:**
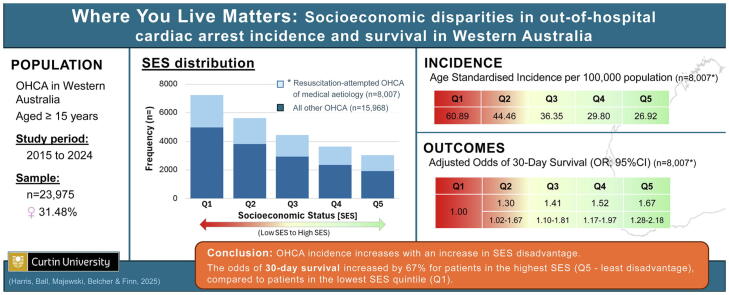

## Acronyms

ABS: Australian Bureau of Statistics
AED: Automated External Defibrillator
BLS: Basic Life Support
CAD: Computer-Aided Dispatch
CPR: Cardiopulmonary Resuscitation
ED: Emergency Department
EMS: Emergency Medical Service
ePCR: Electronic Patient Care Records
IRSD: The ABS Index Relative Socio-Economic Disadvantage
OHCA: Out-Of-Hospital Cardiac Arrest
PRECRU: Prehospital, Resuscitation and Emergency Care Research Unit
ROSC: Return of Spontaneous Circulation
SA1: ABS Statistical Area 1
SEIFA: Australian Bureau of Statistics Socio-Economic Indexes for Areas
SES: Socioeconomic Status
SJ-WA: St John WA
WA: Western Australia

## Introduction

International evidence consistently demonstrates socioeconomic disparities in out-of-hospital cardiac arrest [OHCA],^1^, ^2^, ^3^, ^4^, ^5^ with lower socioeconomic populations experiencing higher incidence and poorer survival.^5^, ^6^, ^7^ However, the magnitude of the effect of socioeconomic status [SES] varies considerably between studies,^5^, ^6^, ^7^, ^8^ possibly reflecting differences in how SES is defined and applied.

Only a small proportion of studies have used composite SES indices that capture multiple aspects of disadvantage.^9^, ^10^, ^11^, ^12^ Many rely on single-domain measures such as income or education,^1^, ^13^, ^14^ which may weight dimensions of SES differently and therefore are not directly comparable across settings.^15^ Studies also differ in their assignment of SES, many using the location of arrest^2^, ^3^, ^10^, ^12^, ^13^, ^16^, ^17^, ^18^ with fewer using patient’s residence.^9^, ^14^, ^19^, ^20^ While some studies assign SES at the individual-level,^21^, ^22^ many use area-level SES values.^2^, ^13^, ^16^, ^23^ For area-level attribution, SES is often assigned using broad geographical units such as local government areas,^17^, ^18^ postcode,^9^, ^16^, ^24^, ^25^, ^26^ census tracts,^1^, ^13^ or districts,^2^, ^10^ where population sizes may exceed 4000 residents.^2^, ^13^, ^27^ When these broader units are combined with other less precise measurements, such as arrest location, the findings may not accurately reflect an individual’s socioeconomic context.^28^, ^29^, ^30^

To our knowledge, only one previous descriptive study^31^ has combined a validated composite SES index with fine spatial resolution and residential address assignment. To address this gap, our study applies the Australian Bureau of Statistics [ABS] Index of Relative Socio-Economic Disadvantage [IRSD]^32^ at the smallest spatial unit available (ABS Statistical Area 1), and uses residential address to approximate individual context – providing a more precise measure of area-level socioeconomic disadvantage for OHCA research.

Examining socioeconomic differences in OHCA incidence and survival within a universal healthcare setting such as Western Australia [WA]^33^ provides an opportunity to explore how structural inequities persist even where financial barriers to care are minimal. Identifying populations at greatest risk is critical for guiding equitable public health policy and targeted strategies to reduce both incidence and mortality.^34^, ^35^ This study investigates OHCA incidence and survival across SES quintiles in WA, providing insights relevant to other regions with comparable health systems.

## Methods

### Study design

We conducted a population-based, retrospective cohort study of OHCA attended by St John WA [SJ-WA] emergency medical services [EMS] in WA between 1st January 2015 and 31st December 2024. Patients were included in the study if at the time of their arrest: (i) their sex was recorded, and they were aged ≥15 years; (ii) their primary residential address was in WA; and (iii) they were not residing at an aged-care facility or correctional facility as the communal nature of these settings means that area-level socioeconomic measures may not accurately reflect individual SES.

### Study setting

WA, the largest Australian state by land area (2.53 million km^2^),^36^ has a total population of approximately 2.98 million.^37^ In WA, SJ-WA is the primary provider of road based EMS,^38^ which operates a single-tiered advanced life support service, staffed in the metropolitan area by nationally registered paramedics, and in rural and remote regions by either paramedic-only crews (offering advanced life support), volunteer emergency medical technicians (offering basic life support only) or hybrid crews. OHCA patients are transported to the closest hospital in rural areas or to one of nine hospital emergency departments within the Perth metropolitan area, unless resuscitative efforts are ceased in the field by SJ-WA (or not commenced), due to futility or pre-existing not-for-resuscitation orders. Therefore all OHCAs attended by EMS within WA in the study time period were captured (including EMS-witnessed OHCAs), with the exception of a portion of the Kimberley, a remote region in the north west of WA with an estimated population of 10,000 people, which is serviced by the Kimberley Ambulance Service, coordinated by the WA Country Health Service.^39^

### Data source

#### OHCA Database

The OHCA Database is maintained by the Prehospital, Resuscitation and Emergency Care Research Unit at Curtin University, WA. The Database is derived from electronic patient care records (completed by ambulance personnel) and computer-aided dispatch information for all SJ-WA attended OHCA arrests. Information includes demographic variables (e.g. age, sex, residential and arrest address), arrest characteristics (e.g. bystander witnessed, bystander-CPR, aetiology, initial arrest rhythm) and EMS details (e.g. EMS response times, EMS defibrillation, and transportation to hospital).

Outcome variables include return of spontaneous circulation [ROSC] on arrival at the hospital Emergency Department and 30-day survival. Thirty-day survival was determined by manual look-up in the WA Death Registry,^40^ for patients not recorded as deceased by SJ-WA EMS personnel.

#### Socioeconomic status

The ABS divides Australia into 61,844 statistical geographical sections (Statistical Area Level 1 [SA1]), each representing approximately 400 individuals per area.^41^ These SA1′s can be aggregated into larger areas: SA2s, SA3s and SA4s. Every five years, the ABS undertakes a population-wide national census, with the geographic classification typically undergoing minor changes between censuses (e.g. SA1s being split due to population growth in those areas). The 2016 and 2021 census versions of classification were used in this study with arrests up to and including 31 December 2019 attributed to the 2016 SA1 classifications and thereafter attributed to the 2021 SA1 classifications.

SES was defined using the Index of Relative Socio-Economic Disadvantage [IRSD] derived from the ABS Socio-Economic Indexes for Areas [SEIFA] based on 2016 and 2021 census data.^32^ The IRSD is a widely used area-level indicator for SES in Australian health research.^18^, ^26^, ^28^, ^29^, ^42^, ^43^, ^44^ The ABS assigns each SA1 area within WA an IRSD state-based decile ranking (1–10) per census period. For this study, the ABS-defined deciles were collapsed to quintile rankings (i.e. deciles 1 & 2 = quintile 1), consistent with common practice to facilitate interpretability, comparability across studies, and statistical stability.^18^, ^26^, ^32^, ^42^ The IRSD quintile ranking (1–5) denotes relative disadvantage, with a lower quintile (Q1) indicating a greater level of SES disadvantage.^45^ A map of the 2021 IRSD SA1 boundaries in WA is provided in the Supplementary Material (S1).

#### Assigning SES status

SES attribution was based on patient residential address, rather than the location of their arrest. For records where residential address was not recorded and where the patient died, we sourced supplementary residential address data through linkage with the WA Death Registry.^40^ Geocodes were determined by address-matching with the Australian Geocoded National Address File.^46^ We used the Geocentric Datum of Australia 1994 for all coordinates. Once geocoding was complete, we imported the OHCA cases as point locations into ArcGIS Desktop v10 (Redlands, California, Environmental Systems Research Institute), together with 2016 and 2021 versions of the ABS geographic boundary files for SA1s.

### Statistical analysis

Data extraction was completed in Microsoft Excel (Microsoft Corporation), and analyses in IBM SPSS Statistics (Version 30.0.0; IBM Corp) and Stata (Release 2019; StataCorp LLC). Descriptive statistics were reported as mean and standard deviation [SD] or median and inter-quartile range [IQR] for continuous variables and frequency (percentage) for categorical variables. Basic inferential comparisons for demographic and arrest characteristics between SES quintiles, used chi-squared (categorical variables), or Kruskal-Wallis tests (continuous variables). Statistical significance was set at *p* < 0.05.

#### Incidence

Crude and age-standardised incidence rates [ASIRs] were calculated over the 10-year period per 100,000 adult population per year. Denominators were defined as total person-years at risk for each five-year period, using the WA population from the 2016 or 2021 Census (for arrests in 2015–2019 and 2020–2024, respectively),^47^, ^48^ stratified by 10-year age group and SES quintile. ASIRs were directly standardised to the 2001 Australian Standard Population.^49^

Negative binomial regression was used to model OHCA incidence as incidence rate ratios [IRR] for (i) all arrests and (ii) resuscitation-attempted arrests of medical aetiology, which we subsequently refer to as the “medical-only sub-cohort’. Each analysis included crude and age/sex-adjusted models, with SES Quintile 1 (lowest SES) as the reference. The log of person-years was included as an offset. Model fit and overdispersion were assessed by comparing Poisson and negative binomial models using Akaike [AIC] and Bayesian [BIC] Information Criteria, dispersion parameter (*α*), and standardised Pearson residuals. Negative binomial models demonstrated lower AIC/BIC values, significant dispersion (*α* > 0, *p* < 0.001), and adequate residual plots, confirming improved model fit.^50^

#### Survival outcome modelling

As OHCAs classified as ‘non-medical’ differ from ‘medical’ OHCAs in aetiology and prognosis,^51^, ^52^ our analysis of survival outcomes was restricted to resuscitation-attempted arrests of medical aetiology (i.e. medical-only sub-cohort). Cases were excluded from the analysis of survival outcomes where resuscitation was not attempted due to a documented ‘Do-Not-Resuscitate’ directive, or where no resuscitation attempt was initiated (either as an EMS resuscitation attempt or where no bystander AED shock was delivered). Logistic regression models were used to examine the association between SES and survival outcomes (ROSC on hospital arrival and 30-day survival). Covariates were pre-specified a priori based on established associations with OHCA prognosis^51^ and entered simultaneously into each model with no stepwise selection or univariate screening performed.^53^ Analyses were conducted within (i) the medical-only sub-cohort and (ii) the medical-only ‘Utstein’ sub-cohort (bystander-witnessed, initial shockable rhythm, EMS-resuscitation attempted or bystander shock delivered).^51^

Three models were constructed per outcome: Model 1 (crude, unadjusted), Model 2 (adjusted for age and sex), and Model 3 (multivariable adjusted). For the medical-only sub-cohort, Model 3 included age (years), sex (male/female), arrest location (public/residential), witness status (unwitnessed/bystander/EMS), bystander CPR (yes/no), initial rhythm (shockable/non-shockable), EMS response time (minutes), and year of arrest. For the Utstein sub-cohort, Model 3 included age, sex, location, bystander CPR, EMS response time, and year. Reference categories were female, private location, unwitnessed, no bystander CPR, and non-shockable rhythm. Model discrimination was assessed using the area under the receiver operating characteristic [ROC] curve for each multivariable adjusted model. Statistical significance was defined as *p* < 0.05.

## Results

### Demographics

**Fig. 1:**
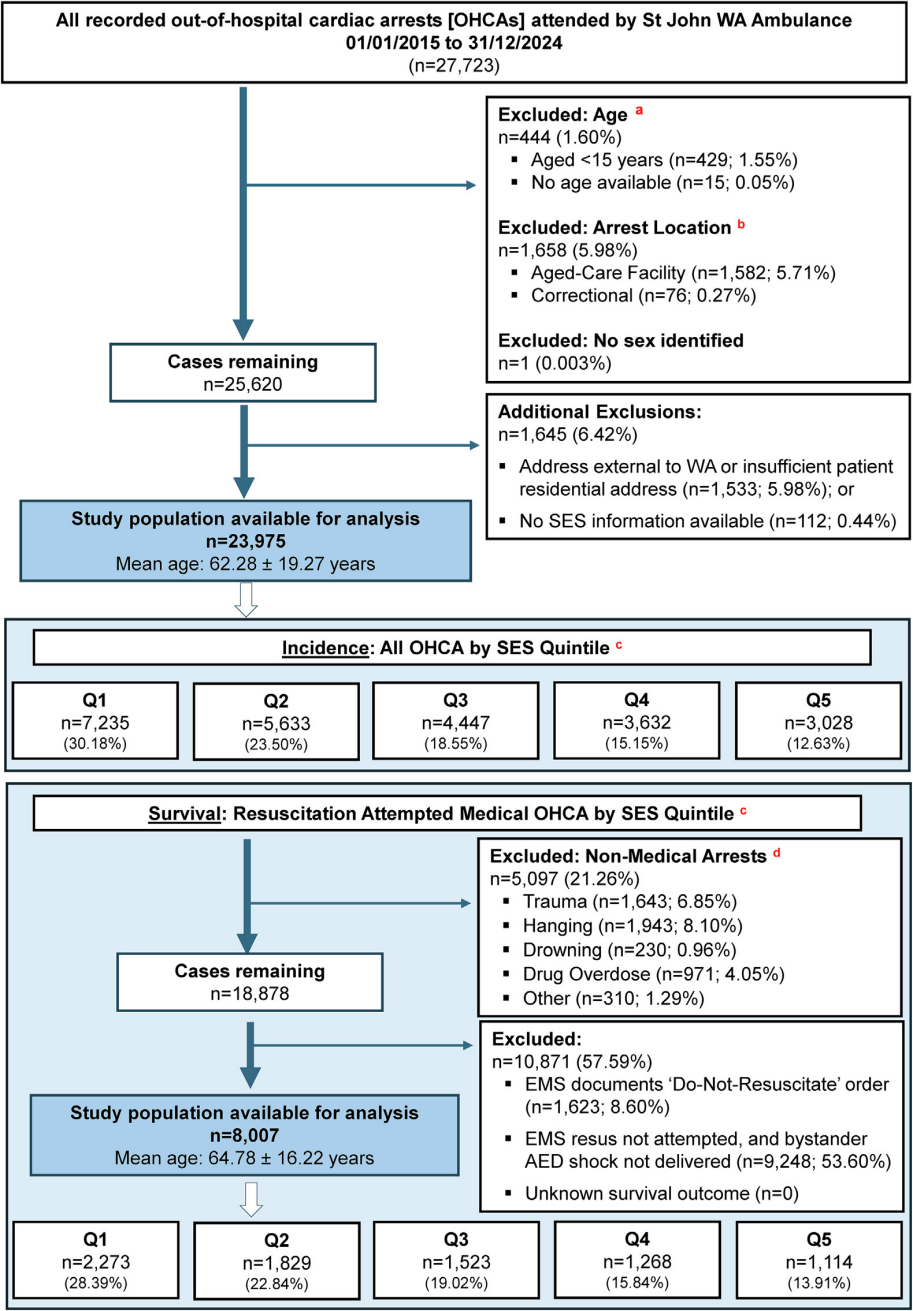
**Sample selection flow-chart**. ^a^Exclusions apply from 15-years of age, as ABS generates data for SES from 15 years of age. ^b^There are no arrests for <15 years at an aged-care facility or correctional facility. ^c^Socioeconomic status [SES] derived from patient’s residential address at time of arrest based on the IRSD at a state-based SA1 level. ^d^Definition of a non-medical arrest.^51^

A total of 27,723 OHCA events were recorded in WA during the 2015 to 2024 period, with 23,975 cases meeting the inclusion criteria (Fig. 1). Of these, 16,428 events were in men (68.52%) (Table 1). The 23,975 included OHCA events were experienced by 23,881 individuals (with 46 patients who had experienced two or more OHCA events during the study period included in the study cohort).

**Table 1:** Characteristics of all EMS attended OHCA in WA, stratified by SES classification quintile (*n* = 23,975).

**Characteristics**	**Socioeconomic status quintiles**						***p*-values**
	**Q1**	**Q2**	**Q3**	**Q4**	**Q5**	**Total**	
Total cases (*n*)	7235 (30.18%)	5633 (23.50%)	4447 (18.55%)	3632 (15.15%)	3028 (12.63%)	23,975 (100.00%)	
Sex, male *n* (%)	4852 (67.06%)	3845 (68.26%)	3065 (68.92%)	2564 (70.59%)	2102 (69.42%)	16,428 (68.52%)	0.003*
Age, median [IQR] (years)	64 [49–76]	64 [48–77]	64 [48–78]	65 [49–78]	68 [51–80]	64 [49–78]	<0.001*
Male, median [IQR] (years)	63[48–75]	62 [46–75]	63 [47–76]	64 [47–76]	66 [50–78]	63 [48–76]	<0.001*
Female, median [IQR] (years)	65[51–79]	68[52–81]	67 [50–81]	70 [51–82]	73 [56–85]	68 [51–81]	<0.001*
**Incidence, per 100,000 adults/year**							
Crude+	193.81	136.90	106.10	85.35	73.18	117.34	<0.001*
Age-standardised++	168.04	125.56	102.27	85.92	71.89	108.87	<0.001*
**Incident region, *n* (%)**							
Rural/remote	2326 (32.15%)	1485 (26.36%)	1037 (23.32%)	665 (18.31%)	273 (9.02%)	5786 (24.13%)	<0.001*
Metro	4909 (67.85%)	4148 (73.64%)	3410 (76.68%)	2967 (81.69%)	2755 (90.98%)	18,189 (75.87%)	
**Incident location, *n* (%)**							
Private residence	6243 (86.29%)	4719 (83.77%)	3680 (82.75%)	2962 (81.55%)	2546 (84.08%)	20,150 (84.05%)	<0.001*
Public place	789 (10.91%)	744 (13.21%)	600 (13.49%)	547 (15.06%)	424 (14.00%)	3104 (12.95%)	
Other	203 (2.81%)	170 (3.02%)	167 (3.76%)	123 (3.39%)	58 (1.92%)	721 (3.01%)	
**Witness status, *n* (%)**							
EMS	407 (5.63%)	339 (6.02%)	286 (6.43%)	226 (6.22%)	202 (6.67%)	1460 (6.09%)	<0.001*
Bystander	1432 (19.79%)	1309 (23.24%)	1023 (23.00%)	892 (24.56%)	776 (25.63%)	5432 (22.66%)	
Unwitnessed	5396 (74.58%)	3985 (70.74%)	3138 (70.56%)	2514 (69.22%)	2050 (67.70%)	17,083 (71.25%)	
#**Bystander CPR (excludes EMS witnessed arrests), *n* (%)**							
Bystander witnessed	945 (13.84%)	881 (16.64%)	710 (17.06%)	636 (18.67%)	553 (19.57%)	3725 (16.54%)	<0.001*
Unwitnessed	1374 (20.12%)	1109 (20.95%)	939 (22.57%)	761 (22.34%)	642 (22.72%)	4825 (21.43%)	
‘Do not resuscitate’ order in place, *n* (%)	458 (6.33%)	393 (6.98%)	287 (6.45%)	250 (6.88%)	253 (8.36%)	1641 (6.84%)	0.005*
Initial shockable arrest rhythm, *n* (%)	586 (8.10%)	535 (9.50%)	470 (10.57%)	460 (12.67%)	393 (12.98%)	2444 (10.19%)	<0.001*
Bystander AED applied, *n* (%)	242 (3.34%)	191 (3.39%)	170 (3.82%)	174 (4.79%)	144 (4.76%)	921 (3.84%)	<0.001*
Bystander AED shock delivered, *n* (%)	87 (1.20%)	81 (1.44%)	81 (1.82%)	84 (2.31%)	62 (2.05%)	395 (1.65%)	<0.001*
**Aetiology, *n* (%)**							
Medical	5536 (76.52%)	4141 (73.51%)	3313 (74.50%)	2614 (71.97%)	2242 (74.04%)	17,846 (74.44%)	<0.001*
Trauma	427 (5.90%)	425 (7.54%)	313 (7.04%)	276 (7.60%)	202 (6.67%)	1643 (6.85%)	
Hanging	557 (7.70%)	441 (7.83%)	380 (8.55%)	329 (9.06%)	236 (7.79%)	1943 (8.10%)	
Drowning	44 (0.61%)	54 (0.96%)	40 (0.90%)	49 (1.35%)	43 (1.42%)	230 (0.96%)	
Drug overdose	268 (3.70%)	245 (4.35%)	177 (3.98%)	155 (4.27%)	126 (4.16%)	971 (4.05%)	
Malignancy	267 (3.69%)	220 (3.91%)	136 (3.06%)	121 (3.33%)	107 (3.53%)	851 (3.55%)	
Other	1336(1.88%)	107(1.90%)	88 (1.98%)	88(2.42%)	72(2.38%)	491 (2.05%)	
EMS resuscitation attempted, *n* (%)	2799 (38.69%)	2303 (40.88%)	1875 (42.16%)	1559 (42.92%)	1358 (44.85%)	9894 (41.27%)	<0.001*
##EMS response time, n	7229	5627	4443	3626	3018	23,943	
Median [IQR] (minutes)	9.48[6.97–13.23]	9.72[7.3–13.7]	9.78[7.23–13.72]	9.73[7.27–13.55]	9.18[6.95–11.9]	9.58[7.13–13.27]	<0.001*
Transport time to hospital, *n* (%)	1515 (20.94%)	1298 (23.04%)	1103 (24.80%)	935 (25.74%)	835 (27.58%)	5686 (23.72%)	
Median [IQR] (minutes)	8.17[4.67–13.37]	10[6.5–13.88]	10.15[6.8–13.77]	10.43[7–14.53]	9.85[6.85–13.83]	9.75[6.08–13.82]	<0.001*
Died at scene, *n* (%)	5716 (79.00%)	4330 (76.87%)	3342 (75.15%)	2696 (74.23%)	2191 (72.36%)	18,275 (76.23%)	<0.001*
**ROSC on hospital arrival, *n* (%)**							
All cases	480 (6.63%)	470 (8.34%)	398 (8.95%)	388 (10.68%)	342 (11.29%)	2078 (8.67%)	<0.001*
**Survival to 30-days, *n* (%)**							
All cases	218 (3.01%)	231 (4.10%)	205 (4.61%)	182 (5.01%)	179 (5.91%)	1015 (4.23%)	<0.001*

SES: Socioeconomic Status; ROSC: Return of Spontaneous Circulation; EMS: Emergency Medical Services; IQR: Interquartile Range; Shockable rhythm includes Ventricular Tachycardia and Ventricular Fibrillation; SES quintiles are defined by the IRSD at an SA1 level. Q1 indicates greatest level of disadvantage (low SES), to Q5 indicating least disadvantage (high SES).

Denominator for all characteristics is *n* = 23,975 unless otherwise specified:

# Bystander CPR excluding EMS witnessed arrests: *n* = 22,515.

## EMS Response Time: *n* = 23,943.

+ Crude incidence rates were calculated using the ABS-defined WA population by IRSD quintile for the corresponding census year (2016, 2021).^47^, ^48^ Differences between SES quintiles were assessed using negative binomial regression.

++ Age-standardised incidence rates were calculated using the ABS-defined Western Australian population by IRSD quintile (2016 and 2021 census years) as the exposure population^47^, ^48^ and standardised to the 2001 Australian Standard Population.^49^ Differences between SES quintiles were assessed using negative binomial regression, adjusting for age in 10-year groups.

* *p*-values significant at the *p* < 0.05 level. Unless otherwise stated, significance is assessed for differences within characteristic categories across the SES quintiles. Significance between groups for age is assessed based on the whole cohort, and separately for males and females.

### Incidence and characteristics of all out-of-hospital cardiac arrests

The overall crude incidence of OHCA in WA was 117.34 per 100,000 adults per year. A clear monotonic socioeconomic gradient was observed, with incidence decreasing from 193.81/100,000 in the most disadvantaged quintile (Q1) to 73.18/100,000 in the least disadvantaged quintile (Q5) (*p* < 0.001; Table 1). The proportion of male and female patients was similar across all quintiles, ranging 67–71% (male), and 29–33% (female) among quintiles.

Most arrests occurred within a private residence (*n* = 20,150; 84.05%) (Table 1), with 90.08% of these private arrests occurring in the person’s own home (*n* = 18,152). Aetiology was similar across SES quintiles for all characteristics and was predominantly presumed cardiac. The proportion of shockable initial cardiac arrest rhythm, EMS attempted resuscitation, bystander witnessed OHCA, bystander CPR and defibrillation significantly increased with increasing SES (*p* < 0.001 for all). The proportion of incidents occurring in rural or remote WA decreased with increasing SES (32.15–9.02%; *p* < 0.001).

**Fig. 2:**
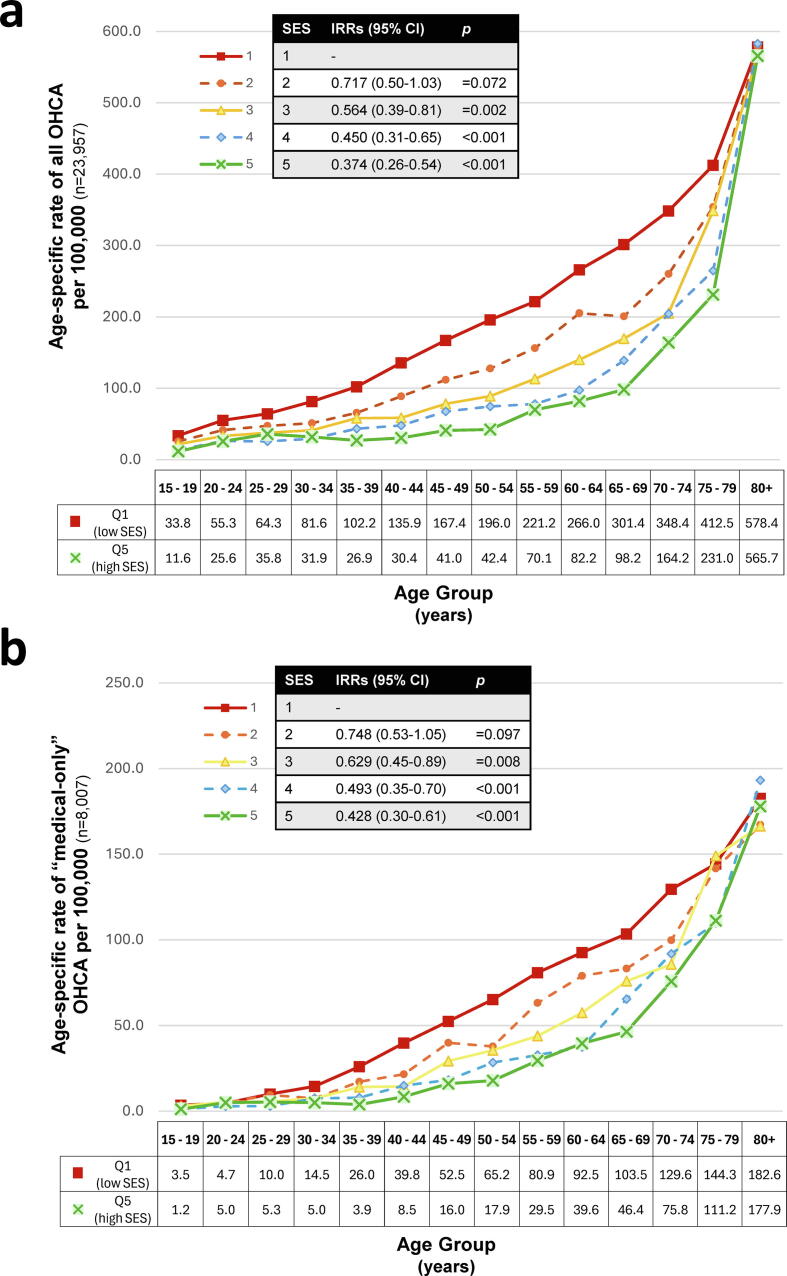
**Age-specific incidence of OHCA by SES quintiles, for the 10-year period between 2015 and 2024**. (a) All OHCA cases (*n* = 23,957). (b) Resuscitation-attempted OHCA of medical aetiology (*n* = 8007). Note the difference in y-axis range between (a) and (b). “Medical OHCA” refers to resuscitation-attempted OHCA of medical aetiology (*n* = 8007). Age-specific incidence rates were calculated per 5-year age-group (years), using the ABS-defined WA population by IRSD quintile (2016 and 2021 census years) as the exposure population and presented per 100,000 adult population per year.^47^, ^48^ Q1 and Q5 denote the lowest (greatest disadvantage, low SES) and highest (least disadvantaged, high SES) SES quintiles, respectively, based on the SEIFA Index of Relative Socio-Economic Disadvantage [IRSD].^32^ Inset: Age-adjusted incidence rate ratios (IRRs) by SES (reference Q1) derived from negative binomial regression, adjusted for age and log of person-years. Statistical significance was set at *p* < 0.05.

Compared with the most disadvantaged quintile (Q1), age-adjusted OHCA incidence was 62.57% lower in the least disadvantaged quintile (Q5) (IRR = 0.37; 95%CI: 0.26–0.54; p < 0.001) (Fig. 2a). Comparing the lowest (Q1) and highest (Q5) SES quintiles, age-specific incidence was most similar at the tail ends of the age spectrum (youngest and oldest groups), where incidence rates differed by less than threefold (Fig. 2a). The disparity widened progressively between ages 35 and 65 years, peaking at ages 40–44 and 50–54 where incidence in Q1 (low SES) was approximately 4.5 times higher than in Q5 (high SES) (Fig. 2a).

### Incidence and characteristics of the medical-only sub-cohort

A total of 8007 OHCA cases (33.40%) were included in the ‘medical-only OHCA” cohort. The overall crude incidence for this sub-cohort was 30.19/100,000 per year. Incidence decreased from 60.89/100,000 in the most disadvantaged quintile (Q1) to 26.92/100,000 in the least disadvantaged quintile (Q5) (*p* < 0.001; Table 2). Men accounted for the majority of events overall (68.69%), with a modest increase in the proportion of males across SES quintiles, from 65.29% in Q1 to 73.07% in Q5 (*p* < 0.001).

**Table 2:** Comparison of resuscitation-attempted OHCAs of medical aetiology in WA, by SES classification quintile (*n* = 8007).

**Characteristics**	**Socioeconomic status quintiles**						***p*-values**
	**Q1**	**Q2**	**Q3**	**Q4**	**Q5**	**Total**	
Total cases, *n* (%)	2273 (28.39%)	1829 (22.84%)	1523 (19.02%)	1268 (15.84%)	1114 (13.91%)	8007 (100.00%)	
Sex, male *n* (%)	1484 (65.29%)	1257 (68.73%)	1044 (68.55%)	901 (71.06%)	814 (73.07%)	5500 (68.69%)	<0.001*
Age, median [IQR] (years)	66 [53–77]	65 [54–77]	66 [53–77]	68 [55–78]	69 [57–79]	67 [54–77]	<0.001*
Male, median [IQR] (years)	66.5[54–77]	65 [54–76]	66 [54–76]	67 [54–77]	68 [57–77]	66 [55–76]	0.005*
Female, median [IQR] (years)	64[52–77]	67[54–79]	66 [52–77]	69 [55–80]	72 [59–84]	67 [53–79]	<0.001*
**Incidence, per 100,000 population**							
Crude+	60.89	44.46	36.35	29.80	26.92	30.19	<0.001*
Age-Standardised++	51.51	40.08	34.55	29.51	25.42	35.63	<0.001*
**Incident region *n* (%)**							
Rural/remote	713 (31.37%)	442 (24.17%)	333 (21.87%)	208 (16.40%)	78 (7.00%)	1774 (22.16%)	<0.001*
Metro	1560 (68.63%)	1387 (75.83%)	1190 (78.14%)	1060 (83.60%)	1036 (93.00%)	6233 (77.84%)	
**Incident location *n* (%)**							
Private residence	1923 (84.60%)	1516 (82.89%)	1244 (81.68%)	1029 (81.15%)	909 (81.60%)	6621 (82.69%)	0.031*
Public place	273 (12.01%)	255 (13.94%)	220 (14.45%)	196 (15.46%)	177 (15.89%)	1121 (14.00%)	
Other	77 (3.39%)	58 (3.17%)	59 (3.87%)	43 (3.39%)	28 (2.51%)	265 (3.31%)	
**Witness status, *n* (%)**							
EMS	312 (13.73%)	250 (13.67%)	211 (13.85%)	164 (12.93%)	144 (12.93%)	1081 (13.50%)	<0.001*
Bystander	973 (42.81%)	871 (47.62%)	695 (45.63%)	621 (48.97%)	540 (48.47%)	3700 (46.21%)	
Unwitnessed	988 (43.47%)	708 (38.71%)	617 (40.51%)	483 (38.09%)	430 (38.60%)	3226 (40.29%)	
#**Bystander CPR (excludes EMS witnessed arrests), *n* (%)**							
Bystander witnessed	731 (37.28%)	677 (42.88%)	554 (42.23%)	514 (46.56%)	438 (45.15%)	2914 (42.07%)	<0.001*
Unwitnessed	674 (34.37%)	488 (30.91%)	459 (34.98%)	353 (31.97%)	316 (32.58%)	2290 (33.06%)	
Initial shockable arrest rhythm, *n* (%)	542 (23.85%)	492 (26.90%)	446 (29.28%)	425 (33.52%)	365 (32.76%)	2270 (28.35%)	<0.001*
Bystander AED applied, *n* (%)	171 (7.52%)	130 (7.11%)	120 (7.88%)	118 (9.31%)	99 (8.89%)	638 (7.97%)	0.144
Bystander AED shock delivered, *n* (%)	85 (3.74%)	75 (4.10%)	75 (4.92%)	79 (6.23%)	54 (4.85%)	368 (4.60%)	0.011*
**Aetiology, *n* (%)**							
Medical: Presumed cardiac	2205 (97.01%)	1773 (96.94%)	1486 (97.57%)	1236 (97.48%)	1073 (96.32%)	7773 (97.08%)	0.650
Medical: Other	68 (2.99%)	56 (3.06%)	37 (2.43%)	32 (2.52%)	41 (3.68%)	234 (2.92%)	
##EMS responses time, *n*	2272	1828	1522	1267	1113	8002	
Median [IQR] (minutes)	10.02[7.38–13.65]	10.01[7.65–13.43]	9.84[7.35–13.33]	9.73[7.4–13.45]	9.32[7.27–11.97]	9.83[7.4–13.22]	<0.001*
Transport time to hospital, *n* (%)	1229 (54.07%)	1040 (56.86%)	915 (60.08%)	770 (60.73%)	683 (61.31%)	4637 (57.91%)	
Median [IQR] (minutes)	8.02[4.62–13.02]	10.00[6.59–13.93]	10.15[6.83–13.77]	10.52[7.23–14.5]	9.92[7–13.58]	9.75[6.15–13.77]	<0.001*
Died at scene, *n* (%)	1041 (45.80%)	787 (43.03%)	606 (39.79%)	497 (39.20%)	430 (38.60%)	3361 (41.98%)	<0.001*
**ROSC on hospital arrival, *n* (%)**							
All cases	378 (16.63%)	371 (20.30%)	336 (22.06%)	328 (25.87%)	292 (26.21%)	1705 (21.30%)	<0.001*
###Utstein comparator group (*n* = 1517)	116 (34.94%)	135 (40.06%)	124 (41.89%)	139 (46.80%)	128 (50.20%)	642 (42.32%)	0.002*
**Survival to 30-days, *n* (%)**							
All cases	183 (8.05%)	198 (10.83%)	184 (12.08%)	174 (13.72%)	164 (14.72%)	903 (11.28%)	<0.001*
###Utstein comparator group (*n* = 1517)	75 (22.59%)	98 (29.08%)	101 (34.12%)	96 (32.32%)	99 (38.82%)	469 (30.92%)	<0.001*

SES: Socioeconomic Status; ROSC: Return of Spontaneous Circulation; EMS: Emergency Medical Services; IQR: Interquartile Range; AED: Automated External Defibrillator. SES quintiles are defined by the IRSD at an SA1 level. Q1 indicates greatest level of disadvantage (low SES), to Q5 indicating least disadvantage (high SES).

Utstein is defined as: Shockable rhythm and bystander witnessed; and either EMS resuscitation attempted, or bystander shock delivered.^51^

Denominator for all characteristics is *n* = 8007 unless otherwise specified:

# Bystander CPR excluding EMS witnessed: *n* = 6926.

## EMS Response Time: *n* = 8002; Transport time to hospital: *n* = 4637.

### ROSC on hospital arrive Utstein: *n* = 1517; Survival to 30-days Utstein: *n* = 1517.

+ Crude incidence rates were calculated using the ABS-defined WA population by IRSD quintile for the corresponding census year (2016, 2021).^47^, ^48^ Differences between SES quintiles were assessed using negative binomial regression.

++ Age-standardised incidence rates were calculated using the ABS-defined Western Australian population by IRSD quintile (2016 and 2021 census years) as the exposure population 47, 48 and standardised to the 2001 Australian Standard Population.^49^ Differences between SES quintiles were assessed using negative binomial regression, adjusting for age in 10-year groups.

* *p*-values significant at the *p* < 0.05 level. Unless otherwise stated, significance is assessed for differences within characteristic categories across the SES quintiles. Significance between groups for age is assessed based on the whole cohort, and separately for males and females. ROSC on hospital arrival; and Survival to 30-days are assessed based on all cases across the SES quintiles, and then for the Utstein comparator group**.**

The proportions of shockable initial arrest rhythm (*p* < 0.001), bystander-witnessed arrest (*p* < 0.001), bystander CPR (*p* < 0.001) and delivery of an AED shock by a bystander (*p* = 0.011) all increased significantly with decreasing socioeconomic disadvantage (Table 2). The proportion of incidents occurring in rural or remote WA decreased significantly with decreasing disadvantage (31.37–7.00%; *p* < 0.001).

Compared with the most disadvantaged quintile (Q1), age-adjusted OHCA incidence was 57.24% lower in the least disadvantaged quintile (Q5) (IRR = 0.43; 95%CI: 0.30–0.61; *p* < 0.001) (Fig. 2b). The greatest disparities were observed between adults aged 35 and 54 years, where incidence in Q1 was approximately 6.67 times higher than Q5 at ages 35–39 years, 4.68 times higher at 40–44 years, and 3.28–3.64 times higher at 45–54 years (Fig. 2b).

### Survival in the medical-only sub-cohort

**Fig. 3:**
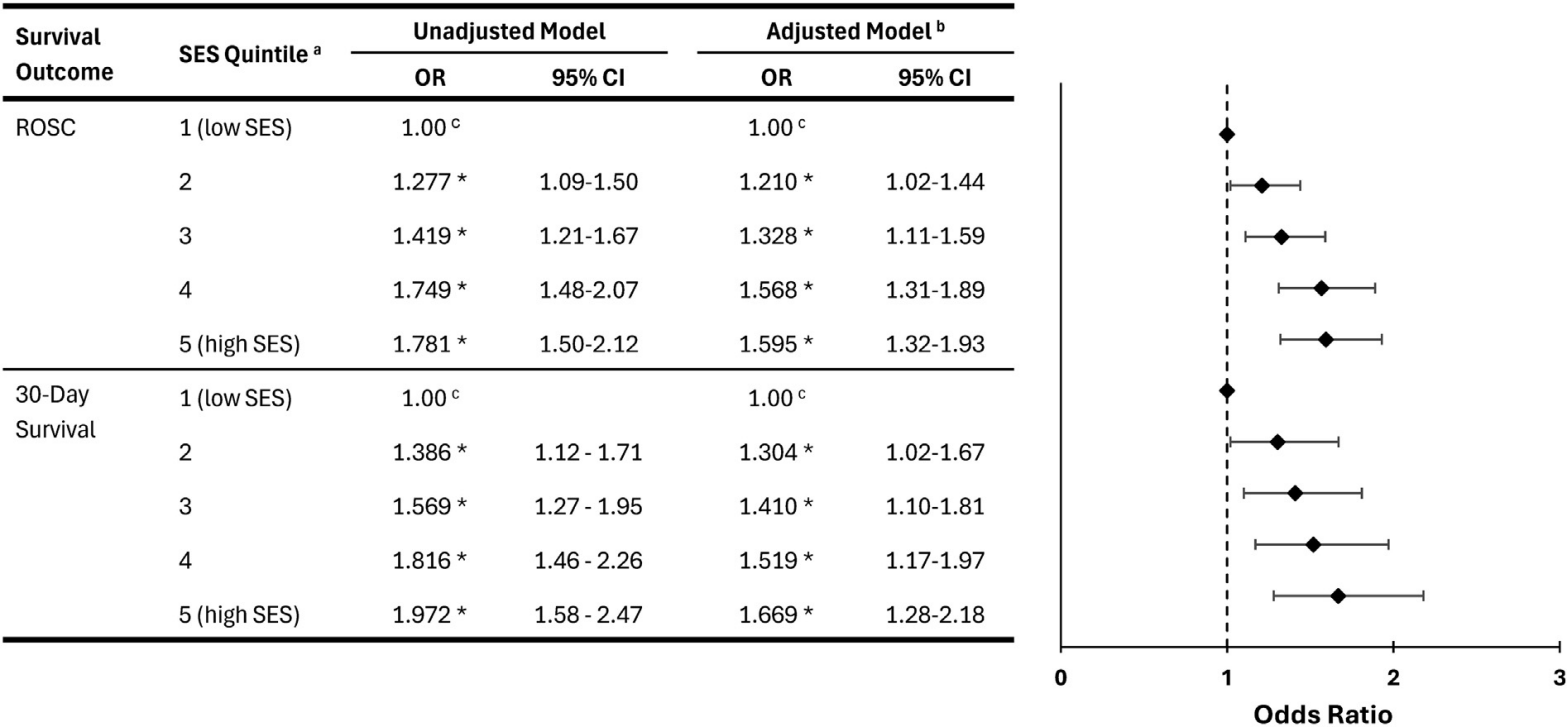
**Effect of socioeconomic status on survival for resuscitation-attempted OHCA of medical aetiology (*n* = 8007)**. SES: Socioeconomic status; OHCA: Out-of-hospital cardiac arrest; ROSC: Return of Spontaneous Circulation on arrival to hospital; OR: Odds Ratio; 95%CI: 95% Confidence Interval. ^a^SES quintiles were derived from the SEIFA Index of Relative Socio-Economic Disadvantage [IRSD] at an SA1 level for Western Australia.^32^ ^b^Adjusted: SES (Quintile 1#); Sex (Female#); Age (years); OCHA Location (Private#); Initial Rhythm (Non-Shockable#); Witnessed OCHA (Unwitnessed#); Bystander CPR (No#); Year of OCHA (years); EMS Time to Respond (minutes). Covariate details are in supplementary material S2a – Model 3. # Denotes categorical variable reference group. ^c^Indicates the reference group. *Note: all SES OR significant at *p* < 0.05.

A monotonic SES gradient was evident, with survival increasing as disadvantage decreased. Among the medical-only sub-cohort, 21.30% achieved ROSC on hospital arrival (*n* = 1705) and 11.28% survived to 30-days (*n* = 903) (Table 2). After adjustment, individuals in the least disadvantaged quintile (Q5) had 1.60-fold higher odds of ROSC (95%CI: 1.32–1.93; *p* < 0.001) and 1.67-fold higher odds of 30-day survival (95%CI: 1.28–2.18; *p* < 0.001) compared with those in the most disadvantaged quintile (Q1) (Fig. 3; supplementary material S3a). Similarly, in the Utstein comparator cohort, Q5 patients had 1.76-fold higher odds of ROSC (95%CI: 1.25–2.50; *p* = 0.001) and double the odds of 30-day survival (OR: 2.14; 95%CI: 1.46–3.14; *p* < 0.001) relative to Q1 (supplementary material S2; supplementary material S3b).

## Discussion

Our study demonstrates a strong socioeconomic gradient in both the incidence and survival of OHCA. Incidence rates were approximately 2.5-fold higher in the most disadvantaged SES quintile (Q1) compared with the least disadvantaged (Q5), while survival showed the reverse pattern. While socioeconomic disadvantage has previously been associated with higher OHCA incidence and poorer survival, the magnitude of this association has varied across studies.^5^, ^6^, ^7^ Our study, which based SES estimation on a small geographical area and used the patient’s residential address, reinforced the consistent association between socioeconomic disadvantage and poorer OHCA outcomes.^2^, ^3^, ^5^, ^7^, ^10^, ^12^, ^17^, ^54^, ^55^, ^56^

This study makes several important contributions to the existing literature on socioeconomic inequalities in OHCA. Using a current, population-based cohort spanning a decade of statewide OHCA data, it provides robust estimates of socioeconomic gradients in both incidence and survival. Additionally, SES was assigned using a granular, small-area geographic unit (SA1) based on the patient’s residential address, reducing misclassification compared with broader area-based measures^28^, ^29^ based on arrest location as used in many prior studies.^18^, ^26^

Specifically, we observed a clear, monotonic increase in both ROSC and 30-day survival with rising SES (adjusted ORs: 1.30–1.67 from Q2–Q5 vs Q1 for 30-days). In contrast, previous Australian studies using broader spatial units reported less consistent gradients.^9^, ^24^ Pemberton et al.^9^ found survival risk ratios that fluctuated across quintiles (RR: 0.89–1.57) before declining in the highest SES group (RR: 0.50; 95%CI: 0.23–1.10), while a later study^24^ reported a similar non-linear pattern for ROSC (RR: 0.88–1.34 from Q2-Q4, to RR: 0.86 in Q5). A New Zealand study applying a composite SES index at fine spatial resolution similarly found no significant trend in ROSC (*p* = 0.84), likely reflecting limited power or population differences.^31^ Earlier work by Clarke et al. identified associations between individual-but not area-level SES when using median income alone.^27^ Collectively, these findings suggest that SES effects may be underestimated when broader or single-domain measures are used, whereas our SA1-level, composite approach perhaps yields more stable and precise estimates of socioeconomic disparities in OHCA outcomes.^28^, ^29^, ^30^, ^31^ Aggregating data across larger or heterogeneous areas likely weakens associations by averaging within-area diversity,^29^ though such effects are unlikely to fully explain the observed differences in gradient shape.

The robustness of the SES gradient after adjustment for age, sex, and key clinical and resuscitation factors (Fig. 3), indicates that socioeconomic disadvantage independently influences outcomes following OHCA. In our study, SES-related differences in both incidence and survival are evident within a universal healthcare system^33^ and operate across the entire socioeconomic continuum, not only among the most disadvantaged. These results align with international evidence showing consistent socioeconomic gradients in OHCA outcomes,^1^, ^2^, ^3^, ^4^, ^5^, ^6^ despite differing healthcare systems and population distributions. That SES gradients are evident despite universal access to healthcare suggests that factors beyond service availability, such as comorbidity burden, health literacy and geographic accessibility, likely contribute to observed inequities in OHCA outcomes.

### Limitations

The findings of this study should be interpretated considering several limitations. Analyses were limited to WA residents with a valid residential address who experienced an OHCA within the study timeframe. However, only 6% of OHCA cases were not WA residents. While SES quintiles were defined using nationally standardised indices, the findings may not be directly generalisable to other Australian jurisdictions or international settings with differing population distributions, healthcare systems, or emergency response models. Cases with missing address data could not be assigned an SES value, which may have introduced exclusion bias; however, this comprised less than 1% of OHCAs. While individual-level measures of SES were not available, SES was assigned using the smallest available geographical unit (SA1) and a composite index of socioeconomic disadvantage. Although area-level SES may not capture individual socioeconomic circumstances, use of smaller-area units such as SA1 reduces misclassification compared with larger geographic measures and improves estimation of socioeconomic gradients in health outcomes.^28^, ^29^ Despite these limitations, the use of a population-based registry, a validated composite SES index and fine scale spatial data provides confidence in the robustness of the observed associations.

### Recommendations

Future research should focus on identifying the unmeasured mechanisms that contribute to socioeconomic disparities in OHCA outcomes.^57^, ^58^ Although this study adjusted for key resuscitation factors, including witness status and bystander CPR,^18^, ^19^, ^23^ SES remained a significant predictor of survival, suggesting that other determinants such as comorbidity burden, health literacy, or access to post-resuscitation care, may contribute to these differences. Understanding these mechanisms will be critical for developing targeted strategies to reduce socioeconomic inequities in OHCA outcomes. Future studies could also examine whether similar SES patterns are evident in paediatric OHCA, where differing aetiologies and social contexts may influence incidence and outcomes.

## Conclusion

This population-based study demonstrates that socioeconomic disparities in OHCA incidence and survival remain evident when SES is measured using a composite index of socioeconomic disadvantage at the smallest available spatial unit derived from individual residential address. The use of this finer-scale, area-based measure of SES revealed a clearer and more consistent gradient than previously reported with broader geographic measures, suggesting that prior variability in effect estimates may partly reflect spatial misclassification. The SES gradient remained evident after multivariable adjustment, indicating that socioeconomic disadvantage independently influences OHCA outcomes. Given the complexities of socioeconomic influences on health, improving the precision of SES measurement will be essential for designing equitable, evidence-based strategies to improve OHCA outcomes.

## Ethics

Ethical approval was granted from Curtin Human Research Ethics Committee (Approval no. HRE2025-0301, as a sub-study to the Western Australian Prehospital Care Record Linkage Project. Approval was also granted by the St John WA Research Governance Committee.

## Declaration of Generative AI and AI-assisted technologies in the writing process

During the preparation of this work, the authors utilised ChatGPT to improve the readability and brevity of the content. After using this tool, the authors reviewed and edited the content as needed and assume full responsibility for the content of the publication.

## CRediT authorship contribution statement

**Sarah Ann Harris:** Writing – review & editing, Writing – original draft, Project administration, Methodology, Formal analysis. **Stephen Ball:** Writing – review & editing, Data curation, Conceptualization. **David Majewski:** Writing – review & editing, Data curation. **Jason Belcher:** Writing – review & editing, Data curation. **Judith Finn:** Writing – review & editing, Project administration, Methodology, Funding acquisition, Data curation, Conceptualization.

## Funding

JF is funded by NHMRC Investigator grant GTN1174838.

## Declaration of competing interest

The authors declare the following financial interests/personal relationships which may be considered as potential competing interests: Professor Judith Finn reports financial support was provided by St John Ambulance Western Australia Ltd. Mr Jason Belcher reports financial support was provided by St John Ambulance Western Australia Ltd. Dr Stephen Ball reports administrative support was provided by St John Ambulance Western Australia Ltd. Professor Judith Finn reports a relationship with National Health and Medical Research Council that includes: funding grants. If there are other authors, they declare that they have no known competing financial interests or personal relationships that could have appeared to influence the work reported in this paper.

## Appendix A Supplementary material

Supplementary material S1 Western Australian 2021 IRSD SA1 boundaries.

Supplementary material S2 Effect of socioeconomic status on survival for resuscitation-attempted OHCA of medical aetiology in an Utstein sub-cohort.

Supplementary material S3 Multivariable analyses Crude; Age and Sex Adjusted; and Multivariable-Adjusted Models. (A) Resuscitation-attempted OHCA of medical aetiology. (B) Utstein, resuscitation-attempted OHCA of medical aetiology.
