# Supplementary material for: Where you live matters: socioeconomic disparities in out-of-hospital cardiac arrest incidence and survival in Western Australia – A population-based cohort study
Source: Resusc Plus. 2026 Feb 18;28:101264. doi: 10.1016/j.resplu.2026.101264 (PMC12969080; doi:10.1016/j.resplu.2026.101264)
Supplement: Supplementary material S1 — Western Australian 2021 IRSD SA1 boundaries. [file mmc1.pdf]

APPENDIX A

Supplementary Material – S1

Western Australian 2021 IRSD SA1 boundaries

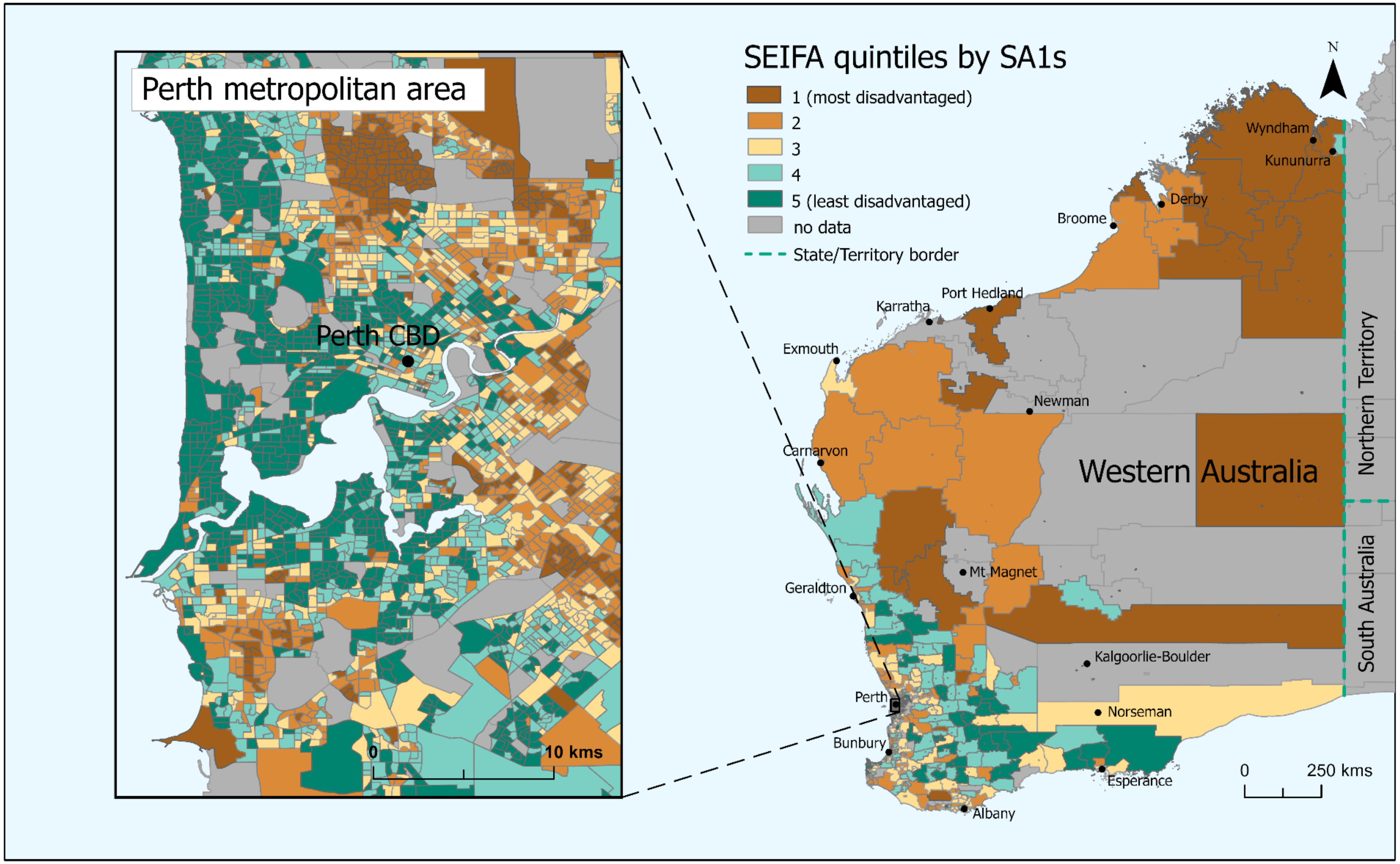

Footnotes:

SES quintiles were derived from the 2021 SEIFA Index of Relative Socio-Economic Disadvantage [IRSD] at an SA1 level for Western Australia. [32]
