# Supplementary material for: Where you live matters: socioeconomic disparities in out-of-hospital cardiac arrest incidence and survival in Western Australia – A population-based cohort study
Source: Resusc Plus. 2026 Feb 18;28:101264. doi: 10.1016/j.resplu.2026.101264 (PMC12969080; doi:10.1016/j.resplu.2026.101264)
Supplement: Supplementary material S2 — Effect of socioeconomic status on survival for resuscitation-attempted OHCA of medical aetiology in an Utstein sub-cohort. [file mmc2.pdf]

APPENDIX A

Supplementary Material – S2

Effect of socioeconomic status on survival for OHCA of a medical aetiology in an Utstein sub-cohort (n=1,517).

Survival Outcome	SES Quintile [a]	Unadjusted Model		Adjusted Model [b]	
		OR	95% CI	OR	95% CI
ROSC	1 (low SES)	1.00 [c]		1.00 [c]	
	2	1.244	0.91-1.70	1.127	0.81-1.56
	3	1.342	0.97-1.85	1.226	0.88-1.72
	4	1.638 *	1.19-2.26	1.530 *	1.09-2.14
	5 (high SES)	1.877 *	1.35-2.62	1.764 *	1.25-2.50
30-Day Survival	1 (low SES)	1.00 [c]		1.00 [c]	
	2	1.405	0.99 - 1.99	1.206	0.83-1.75
	3	1.775 *	1.25 - 2.52	1.595 *	1.10-2.32
	4	1.637 *	1.15 - 2.33	1.522 *	1.05-2.22
	5 (high SES)	2.175 *	1.52 - 3.12	2.139 *	1.46-3.14

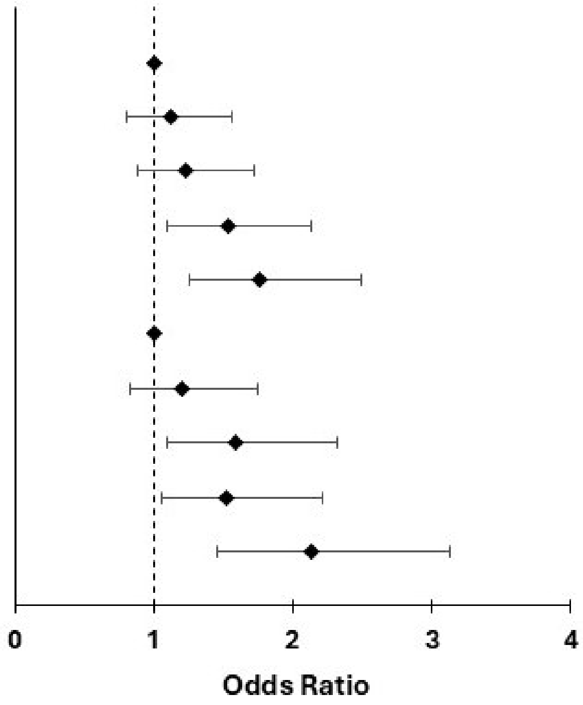

Footnotes:

SES: Socio-economic status; OHCA: Out-of-hospital cardiac arrest; ROSC: Return of Spontaneous Circulation on arrival to hospital; OR: Odds Ratio; 95%CI: 95% Confidence Interval. Utstein is defined as: Shockable rhythm and bystander witnessed; and either EMS resuscitation attempted or bystander shock delivered. [51]

[a] SES quintiles were derived from the SEIFA Index of Relative Socio-Economic Disadvantage [IRSD] at an SA1 level for Western Australia. [32]

[b] Adjusted: SES (Quintile 1#); Sex (Female#); Age (years); OHCA Location (Private#); Bystander CPR (No#); Year of OHCA (years); EMS Time to Respond (minutes). Covariate details are in supplementary material S3b– Model 3. # Denotes categorical variable reference group.

[c] Indicates the reference group.

* Significant at $p<0.05$.
