# Supplementary material for: Where you live matters: socioeconomic disparities in out-of-hospital cardiac arrest incidence and survival in Western Australia – A population-based cohort study
Source: Resusc Plus. 2026 Feb 18;28:101264. doi: 10.1016/j.resplu.2026.101264 (PMC12969080; doi:10.1016/j.resplu.2026.101264)
Supplement: Supplementary material S3 — Multivariable analyses Crude; Age and Sex Adjusted; and Multivariable-Adjusted Models. (A) Resuscitation-attempted OHCA of medical aetiology. (B) Utstein, resuscitation-attempted OHCA of medical aetiology. [file mmc3.pdf]

APPENDIX A

Supplementary Material S3a

Multivariable analyses of resuscitation-attempted, medical aetiology OHCA by SES classification quintile – Crude; Age and Sex Adjusted; and Multivariable-Adjusted Models (n=8,007).

**Model 1: Crude [a]**				
**Factor**	**ROSC on arrival at hospital**		**30-Day Survival**	
	**Unadjusted OR (95% CI)**	***p* value**	**Unadjusted OR (95% CI)**	***p* value**
**SES**				
Q1 (Low SES)	1.00		1.00	
Q2	1.277 (1.09-1.50)	0.003 *	1.386 (1.12-1.71)	0.002 *
Q3	1.419 (1.21-1.67)	<0.001 *	1.569 (1.27-1.95)	<0.001 *
Q4	1.749 (1.48-2.07)	<0.001 *	1.816 (1.46-2.26)	<0.001 *
Q5 (High SES)	1.781 (1.50-2.12)	<0.001 *	1.972 (1.58-2.47)	<0.001 *

**Model 2: Age and Sex Adjusted [b]**				
**Factor**	**ROSC on arrival at hospital**		**30-Day Survival**	
	**OR (95% CI)**	***p* value**	**OR (95% CI)**	***p* value**
**SES**				
Q1 (Low SES)	1.00		1.00	
Q2	1.277 (1.09-1.50)	0.003 *	1.366 (1.11-1.69)	0.004 *
Q3	1.420 (1.21-1.67)	<0.001 *	1.554 (1.25-1.93)	<0.001 *
Q4	1.768 (1.50-2.09)	<0.001 *	1.835 (1.47-2.29)	<0.001 *
Q5 (High SES)	1.816 (1.53-2.16)	<0.001 *	2.024 (1.61-2.54)	<0.001 *
**Age (years)**	0.994 (0.99-1.00)	<0.001 *	0.982 (0.98-0.99)	<0.001 *
**Sex**				
Female	1.00		1.00	
Male	0.980 (0.87-1.10)	0.734 *	1.419 (1.21-1.67)	<0.001 *

**Model 3: Multivariable-Adjusted** [c]				
**Factor**	**ROSC on arrival at hospital**		**30-Day Survival**	
	**OR (95% CI)**	***p* value**	**OR (95% CI)**	***p* value**
**SES**				
Q1 (Low SES)	1.00		1.00	
Q2	1.210 (1.02-1.44)	0.030 *	1.304 (1.02-1.67)	0.034 *
Q3	1.328 (1.11-1.59)	0.002 *	1.410 (1.10-1.81)	0.007 *
Q4	1.568 (1.31-1.89)	<0.001 *	1.519 (1.17-1.97)	0.001 *
Q5 (High SES)	1.595 (1.32-1.93)	<0.001 *	1.669 (1.28-2.18)	<0.001 *
**Age (years)**	0.994 (0.99-1.00)	<0.001 *	0.979 (0.97-0.98)	<0.001 *
**Sex**				
Female	1.00		1.00	
Male	0.763 (0.67-0.87)	<0.001 *	0.999 (0.83-1.21)	0.989
**Location**				
Private	1.00		1.00	
Public	1.378 (1.18-1.62)	<0.001 *	1.942 (1.60-2.36)	<0.001 *
Other	1.375 (1.01-1.87)	0.043 *	1.828 (1.24-2.69)	0.002 *
**Initial Rhythm**				
Non-Shockable	1.00		1.00	
Shockable	3.769 (3.32-4.28)	<0.001 *	11.992 (9.85-14.60)	<0.001 *
**Witnessed OHCA**				
EMS Witnessed	6.538 (5.27-8.11)	<0.001 *	15.606 (11.12-21.90)	<0.001 *
Witnessed	2.814 (2.41-3.28)	<0.001 *	2.746 (2.13-3.55)	<0.001 *
Unwitnessed	1.00		1.00	
**Bystander CPR**				
Yes	1.090 (0.93-1.28)	0.295	1.262 (0.98-1.63)	0.074
No	1.00		1.00	
**Year of OHCA**	1.002 (0.98-1.02)	0.874	0.975 (0.95-1.00)	0.081
**EMS Response Time (minutes)**	0.965 (0.96-0.98)	<0.001 *	0.956 (0.94-0.97)	<0.001 *

Footnotes:

SES: Socio-economic status; ROSC: Return of spontaneous circulation; OHCA: out-of-hospital cardiac arrest; OR: Odds Ratio; Q: SES quintile 1 (low SES) and SES quintile 5 (high SES)

SES quintiles were derived from the WA SEIFA Index of Relative Socio-Economic Disadvantage (IRSD) at the SA1 level, with Q1 indicating greatest disadvantage (low SES) and Q5 least disadvantage (high SES). [32]

[a] Model 1: No adjustments

[b] Model 2: Adjusted for SES (Quintile 1#); Sex (Female#); Age (years)

[c] Model 3: Adjusted for SES (Quintile 1#); Sex (Female#); Age (years); OHCA Location (Private#); Initial Rhythm (Non-shockable#); Witnessed (Unwitnessed#); Bystander CPR (No#); Year of OHCA (years); EMS Time to Respond (minutes)

# Denotes categorical variable reference group

* Logistic regression model significant at p<0.05

Supplementary Material – S3b

Multivariable analyses of Utstein, resuscitation-attempted, medical aetiology OHCA by SES classification quintile – Crude; Age and Sex Adjusted; and Multivariable-Adjusted Models (n=1,517)

Model 1: Crude [a]				
**Factor**	**ROSC on arrival at hospital**		**30-Day Survival**	
	**Unadjusted OR (95% CI)**	***p* value**	**Unadjusted OR (95% CI)**	***p* value**
**SES**				
Q1 (Low SES)	1.00		1.00	
Q2	1.244 (0.91-1.70)	0.172	1.405 (0.99-1.99)	0.056
Q3	1.342 (0.97-1.85)	0.074	1.775 (1.25-2.52)	0.001 *
Q4	1.638 (1.19-2.26)	0.003 *	1.637 (1.15-2.33)	0.006 *
Q5 (High SES)	1.877 (1.35-2.62)	<0.001 *	2.175 (1.52-3.12)	<0.001 *

Model 2: Age and Sex Adjusted [b]				
**Factor**	**ROSC on arrival at hospital**		**30-Day Survival**	
	**Adjusted OR (95% CI)**	***p* value**	**Adjusted OR (95% CI)**	***p* value**
**SES**				
Q1 (Low SES)	1.00		1.00	
Q2	1.243 (0.91-1.70)	0.176	1.392 (0.98-1.98)	0.068
Q3	1.354 (0.98-1.87)	0.067	1.781 (1.24-2.55)	0.002 *
Q4	1.678 (1.22-2.32)	0.002 *	1.716 (1.20-2.46)	0.003 *
Q5 (High SES)	1.958 (1.40-2.74)	<0.001 *	2.405 (1.66-3.48)	<0.001 *
**Age (years)**	0.991 (0.98-1.00)	0.012 *	0.974 (0.97-0.98)	<0.001 *
**Sex**				
Female	1.00		1.00	
Male	0.894 (0.69-1.15)	0.386	1.319 (0.99-1.76)	0.059

**Model 3: Multivariable-Adjusted [c]**				
**Factor**	**ROSC on arrival at hospital**		**30-Day Survival**	
	**Adjusted OR (95% CI)**	***p* value**	**Adjusted OR (95% CI)**	***p* value**
**SES**				
Q1 (Low SES)	1.00		1.00	
Q2	1.127 (0.81-1.56)	0.473	1.206 (0.83-1.75)	0.322
Q3	1.226 (0.88-1.72)	0.237	1.595 (1.10-2.32)	0.015 *
Q4	1.530 (1.09-2.14)	0.013 *	1.522 (1.05-2.22)	0.029 *
Q5 (High SES)	1.764 (1.25-2.50)	0.001 *	2.139 (1.46-3.14)	<0.001 *
**Age (years)**	0.990 (0.98-1.00)	0.004 *	0.971 (0.96-0.98)	<0.001 *
**Sex**				
Female	1.00		1.00	
Male	0.828 (0.64-1.08)	0.164	1.206 (0.89-1.63)	0.224
**Location**				
Private	1.00		1.00	
Public	1.727 (1.37-2.18)	<0.001 *	2.427 (1.89-3.11)	<0.001 *
Other	2.320 (1.40-3.84)	0.001 *	2.357 (1.39-3.99)	0.001 *
**Year of OHCA**	1.019 (0.98-1.06)	0.336	0.999 (0.96-1.04)	0.975
**Bystander CPR**				
Yes	2.048 (1.42-2.94)	<0.001 *	2.354 (1.51-3.67)	<0.001 *
No	1.00		1.00	
**EMS Response Time (minutes)**	0.916 (0.89-0.94)	<0.001 *	0.913 (0.89-0.94)	<0.001 *

Footnotes:

SES: Socio-economic status; ROSC: Return of spontaneous circulation; OHCA: out-of-hospital cardiac arrest; OR: Odds Ratio; Q: SES quintile 1 (low SES) and SES quintile 5 (high SES)

SES quintiles were derived from the WA SEIFA Index of Relative Socio-Economic Disadvantage (IRSD) at the SA1 level, with Q1 indicating greatest disadvantage (low SES) and Q5 least disadvantage (high SES).[32]

Utstein is defined as: Shockable rhythm and bystander witnessed; and either EMS resuscitation attempted or bystander shock delivered.[51]

[a] Model 1: No adjustments

[b] Model 2: Adjusted for SES (Quintile 1#); Sex (Female#); Age (years)

[c] Model 3: Adjusted for SES (Quintile 1#); Sex (Female#); Age (years); OHCA Location (Private#); Bystander CPR (No#); Year of OHCA (years); EMS Time to Respond (minutes)

# Denotes categorical variable reference group

* Logistic regression model significant at $p<0.05$

Appendix A Supplementary data

Supplementary Material S1

Western Australian 2021 IRSD SA1 boundaries

Supplementary Material S2

Effect of socioeconomic status on survival for resuscitation-attempted OHCA of medical aetiology in an Utstein sub-cohort.

Supplementary Material S3

Multivariable analyses Crude; Age and Sex Adjusted; and Multivariable-Adjusted Models. (A) – Resuscitation-attempted OHCA of medical aetiology. (B) – Utstein, resuscitation-attempted OHCA of medical aetiology.
